# Exercising in Air Pollution: The Cleanest versus Dirtiest Cities Challenge

**DOI:** 10.3390/ijerph15071502

**Published:** 2018-07-17

**Authors:** Leonardo Alves Pasqua, Mayara Vieira Damasceno, Ramon Cruz, Monique Matsuda, Marco Garcia Martins, Adriano Eduardo Lima-Silva, Mônica Marquezini, Paulo Hilário Nascimento Saldiva, Romulo Bertuzzi

**Affiliations:** 1 Endurance Performance Research Group (GEDAE-USP), School of Physical Education and Sport, University of São Paulo, São Paulo 05508030, Brazil; damascenomay@gmail.com (M.V.D.); ramonzepp@yahoo.com.br (R.C.); bertuzzi@usp.br (R.B.); 2 Laboratory of Investigation in Ophthalmology (LIM-33), Division of Ophthalmology, University of São Paulo Faculty of Medicine, São Paulo 01246903, Brazil; moniquematsuda@yahoo.com.br; 3 School of Public Health, Harvard University, Boston, MA 02115, USA; marcogarciamartins@gmail.com; 4 Human Performance Research Group, Academic Department of Physical Education (DAEFI), Technological Federal University of Parana, Curitiba 80230901, Brazil; limasilvaae@hotmail.com; 5 Laboratory of Experimental Air Pollution, Department of Pathology, University of São Paulo Faculty of Medicine, São Paulo 01246903, Brazil; marquezinissa@uol.com.br (M.M.); pepino@usp.br (P.H.N.S.); 6 Pro-Sangue Foundation, São Paulo 01246903, Brazil; 7 Institute of Advanced Studies, University of São Paulo, São Paulo 01246903, Brazil

**Keywords:** exercise, air pollution, health, environmental justice

## Abstract

*Background*: Aerobic exercise is recommended to improve health. However, the increased ventilation might increase the doses of inhaled air pollutants, negating the health benefits in highly polluted areas. Our objective was to estimate the inhaled dose of air pollutants during two simulated exercise sessions at cleanest and dirtiest cities reported by World Health Organization (WHO) considering air quality. *Methods*: Minute ventilation data were extracted from laboratory-based exercise of 116 incremental running tests and used to calculate total ventilation of a hypothetical 30-min moderate continuous exercise routine. Afterwards, total ventilation values were combined with particulate matter (PM) data reported by the WHO for the 10 cleanest and 10 dirtiest cities, to calculate inhaled doses and the relative risk of all-cause mortality by exercising in different air pollution concentrations. *Findings*: The dirtiest cities are located at less developed countries compared to cleanest cities. The inhaled dose of PM_2.5_ and PM_10_ were significantly higher in the dirtiest cities compared to the cleanest cities at rest and exercise, and significantly higher during exercise compared to the rest at dirtiest cities. The relative risk of all-cause mortality analysis showed that, while exercise in the cleanest cities improved health benefits throughout up to 90 min, there were no further health benefits after 15 min of exercise in the dirtiest cities, and the air pollution health risks surpassed the exercise benefits after 75 min. *Interpretation*: Our findings suggest that a traditional 30-min of moderate aerobic exercise session might induce inhalation of high levels of pollutants when performed at dirtiest cities. Considering several adverse health effects from air pollutants inhalation, so the results suggest that the air pollution levels of the cities should be taken into account for physical exercise recommendations.

## 1. Introduction

Aerobic exercise has been pointed out as one of the most efficient non-pharmacological practices for improvements in health and life quality, presenting evidence of efficacy in controlling some of the highest death risks, such as hypertension [1] and obesity [2]. The Official American College of Sports Medicine (ACSM) exercise position recommends regular physical activity for health maintenance [3]. It has been suggested that at least 30 min of continuous moderate intensity aerobic exercise (~65% of the maximal oxygen uptake (${\mathrm{VO}}_{2}\max$)) should be performed from three to five days a week [3]. These recommendations have been made on basis of extensive reports showing that exercise is able to promote positive morpho-physiological adaptations in the cardiorespiratory system [4].

Although the abovementioned studies have provided relevant information about the importance of aerobic exercise to improvement of human health, it is important to note that some environmental characteristics are able to modulate exercise benefits, such as air pollution [5]. Air pollution has been considered one of the great killers in our age [6]. According to The State of Global Air 2017, about 90% of people have lived in cities which exceed the air pollution limits established by World Health Organization (WHO). This has led the air pollution to become an important research topic as discussed at the Third UN Conference on Housing and Sustainable Urban Development (Habitat III), Quito, Ecuador, in October 2016 [7]. Previous findings have suggested that short-term exposure to air pollution might cause cardiovascular failures, increase respiratory hospitalization and oxidative stress damage, while long-term air pollution exposure might cause cardiovascular mortality, cancer, premature death [8], and asthma [9]. It is particularly relevant because during aerobic exercise the higher minute ventilation (VE) could lead to a higher inhalation of pollutants [5], what has been already discussed in previous studies and analyzed with different methods by Dons et al. [10]. One of the first studies directly investigating the association between the increased VE during exercise and air pollutants inhalation was conducted by Panis et al. [11]. These authors observed that, despite distinct concentrations between different locations, cyclists presented a VE 4.3 times higher compared to car passengers on average. These higher values of VE might be determinant to higher air pollutants inhalation during active transportation compared to driving in a car. 

Among the main existent air pollutants, particulate matter (PM) has been extensively investigated and has been shown to have a dangerous impact on health [12]. PM are extremely small solid and liquid suspended particles, and have been defined as coarse (PM_10_) and fine (PM_2.5_). For instance, Pekkanen et al. [13] observed a higher risk of ST-segment depression during exercise at higher levels of PM_2.5_. It is believed that, depending on its aerodynamic diameter, PM is able to be deposited at different locals in the respiratory system, leading to local inflammatory processes [12]. Furthermore, for the smallest particles, PM is able to cross alveolar barrier, reaching the blood circulation and leading to cardiovascular and systemic losses [12]. 

In this sense, a dilemma is established, because the positive effects of exercise could be suppressed by air pollution. Recently, the WHO reported a wide variation in air pollution among different cities across the world [14]. Thus, exercising in these different cities could trigger distinct effects due to the high difference among inhaled pollutants. For instance, Laeremans et al. [15] observed and increased sympathetic tone related to exercise and black carbon exposure. Interestingly, this study also showed a decrease in lung function associated to increased black carbon exposure, while was indicated a potential protective effect of exercise. Besides these important advances in understanding air pollution impacts on human health, no studies have demonstrated if the basic exercise recommendations might result in different pollutants inhaled dose when performed under distinct air pollution levels. Therefore, the main objective of this study was to compare the PM_10_ and PM_2.5_ inhaled dose during a traditional 30 min daily exercise recommendation at 10 cleanest and dirtiest cities related to PM annual concentrations reported by WHO [14]. Our hypothesis was that exercise at dirtiest cities could elevate the inhaled doses of air pollutants at extremely higher levels compared to the cleanest cities.

## 2. Methods

### 2.1. Experimental Design

Healthy male volunteers (116) were recruited for participation in the present study (age 25 ± 4 years; body mass 77.8 ± 13.9 kg; height 173 ± 21 cm). They performed a maximal incremental running test on a treadmill. The tests were performed in the city of Sao Paulo (Brazil) at the same time of day in a controlled temperature room (20–24 °C), 2–3 h after the last meal. All individuals were asked to refrain from any exhaustive or unaccustomed exercise for the 48 h preceding the test. They were also instructed to wear standard running shoes and were not taking nutritional supplements at least three months before the study. From the incremental tests, only the VE data were extracted to predict total ventilation (VE_TOTAL_) of two 30-min hypothetical situations: rest and continuous moderate exercise. After the estimation of the VE_TOTAL_ for 30 min of rest and exercise, the inhaled PM_10_ and PM_2.5_ were calculated for each considered city, accordingly to the WHO report of air pollution data 2016 [14]. All experimental procedures were previously approved by the Ethics Committee for Human Studies from the School of Physical Education and Sport of University of São Paulo (2010/44). 

### 2.2. Maximal Incremental Running Test

After a 3-min rest and a 3-min warm-up at 8 km·h^−1^, the treadmill speed (model TK35, CEFISE, Nova Odessa, Brazil) was increased by 1 km·h^−1^ every minute until exhaustion. Gas exchanges were measured breath-by-breath at rest and during the test using a gas analyzer (Cortex Metalyzer 3B, Cortex Biophysik, Leipzig, Germany). Data were subsequently averaged over 30 s intervals throughout the test. Before each test, the gas analyzer was calibrated according to the manufacturer’s recommendations. After the maximal incremental running tests, VE data were extracted and used to predict VE_TOTAL_ in two hypothetical 30-min periods: rest and 65% of the VO_2_max. In turn, VE_TOTAL_ was used to calculate air pollutants inhaled dose, as described below.

### 2.3. Ventilation and Air Pollutants Inhaled Dose Calculations

In order to predict the ventilation related with an exercise session according to ACSM recommendations [3], the rest and maximal incremental test data were used. The VE data (L·min^−1^) from incremental tests were used to calculate the total ventilation (VE_TOTAL_ (L)) for the two hypothetical conditions: 30-min rest, and 30-min continuous exercise at 65% of the VO_2_max. This intensity was chosen based on moderate intensity aerobic exercise recommendations [3], once different exercise intensities might result in distinct VE values. Thus, the VE corresponding to 65% of the VO_2_max was extracted from incremental test and used in Equation (1):

VE_TOTAL_ (L) = VE (L·min^−1^) × 30 min (1)

where VE_TOTAL_ is the total ventilation during the exercise; VE is the minute ventilation; and 30 is the total time in minutes (exercise or rest) chosen for each situation to calculate inhaled dose. Further, the VE_TOTAL_ values were used to calculate the inhaled PM_10_ and PM_2.5_ at each situation [16], according to Equation (2).

PM_INHALED_ (µg) = VE_TOTAL_ × PM_CONC_ (µg·m^−3^)/1000 (2)

where PM_INHALED_ is the total PM_10_ or PM_2.5_ inhaled during each situation, PM_CONC_ is the PM concentration at each ranked city [14] and VE_TOTAL_ is the total ventilation during the considered period.

### 2.4. Air Pollution Data

The air pollution data were selected from the datasheet published in 2016 by the WHO (http://www.who.int/phe/health_topics/outdoorair/databases/en/; WHO_AAP_database_May2016_v3web). The inclusion criterion was that the annual mean concentration of PM_10_ or PM_2.5_ should be measured instead estimated in the WHO database. Then, the 10 cities with the lowest levels and the 10 cities with the highest levels of measured PM_10_ and PM_2.5_ were chosen for analysis of the inhaled doses of these pollutants.

### 2.5. Tipping Point and Break-Even Point Determination

The reduction in the relative risk of all-cause mortality, first used by de Hartog et al. [17] has been analyzed as described by Tainio et al. [18] for running at cleanest and dirtiest cities. The time spent running was converted to metabolic equivalent of task and the risk reduction was calculated using dose-response functions. In combination, the health risks of PM inhalation during exercise were estimated using PM concentrations and the VE during each exercise type. Additionally, it was estimated the tipping point (i.e., additional exercise duration will no longer lead to an increase in health benefits) and the break-even point (i.e., the risks of air pollution starts outweighing the exercise benefits) for the different exercise situations. For more detailed information and calculation spreadsheet, see supplementary material available by Tainio et al. [18]. 

### 2.6. Statistical Analysis

Data normality was assessed through the Shapiro-Wilk test. The comparison between inhaled PM_10_ and PM_2.5_ between cities (cleanest and dirtiest) and situations (rest and exercise) was performed with a two-way ANOVA. All analyses were conducted using the SPSS software (version 17.0, SPSS Inc., Chicago, IL, USA), and the significance level was set at α = 0.05.

## 3. Results

### 3.1. Cleanest and Dirtiest Cities Air Pollution Levels

**Figure 1:**
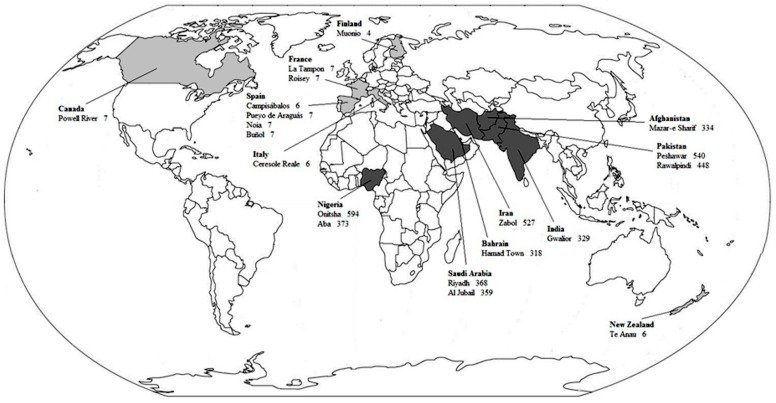
PM_10_ annual means by the 10 cleanest and 10 dirtiest cities. Countries with the cleanest cities are in light gray. Countries with the dirtiest cities are in dark gray. Adapted from the WHO data on the average annual concentration of PM_10_ per city (data from http://www.who.int/phe/health_topics/outdoorair/databases/en/) [14].

**Figure 2:**
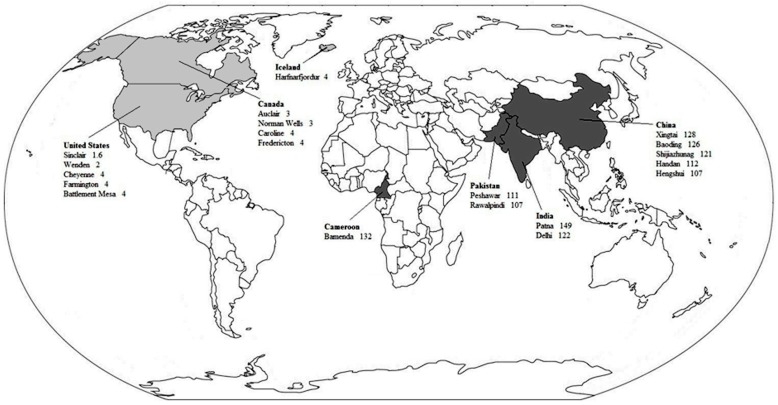
PM_2.5_ annual means by the 10 cleanest and 10 dirtiest cities. Countries with the cleanest cities are in light gray. Countries with the dirtiest cities are in dark gray. Adapted from the WHO data on the average annual concentration of PM_2.5_ per city (data from http://www.who.int/phe/health_topics/outdoorair/databases/en/) [14].

The levels of PM_10_ and PM_2.5_ for each chosen city are presented in Figure 1 and Figure 2, respectively. The levels of PM_10_ and PM_2.5_ in the dirtiest cities were approximately 47 and 34 times higher than in the cleanest cities, respectively.

### 3.2. Calculated Minute Ventilation and Total Ventilation

Calculated ventilations for rest and exercise are presented in Table 1. The VE and VE_TOTAL_ were significantly higher in exercise when compared to rest (*p* < 0.001).

### 3.3. Inhaled Particulate Matter

**Figure 3:**
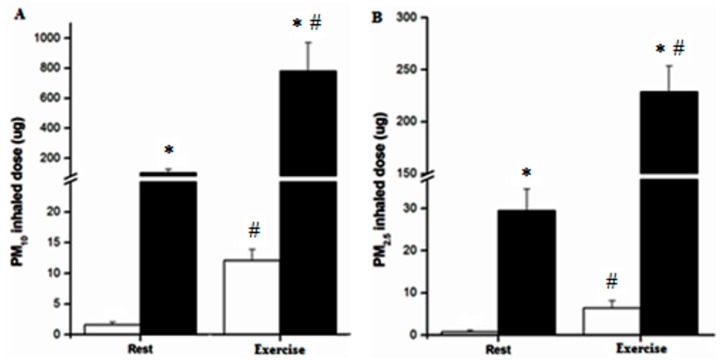
Inhaled PM_10_ (panel (**A**)) and PM_2.5_ (panel (**B**)) during 30 min at rest and exercise by city category: cleanest (white bars) and dirtiest (black bars). * Significantly different from rest at the same city category (cleanest or dirtiest); # Significantly different from the same situation (rest or exercise) at different city category (*p* < 0.05).

Figure 3 shows the estimated inhaled PM_10_ and PM_2.5_ during 30 min at rest and exercise in the dirtiest and cleanest cities. The inhaled PM_10_ and PM_2.5_ were significantly higher for rest and exercise at dirtiest compared to cleanest cities (*p* < 0.001). The inhaled PM_10_ and PM_2.5_ were significantly higher during exercise when compared to rest at each city category (*p* < 0.001).

### 3.4. Relative Risk of All-Cause Mortality for Running at Cleanest and Dirtiest Cities

**Figure 4:**
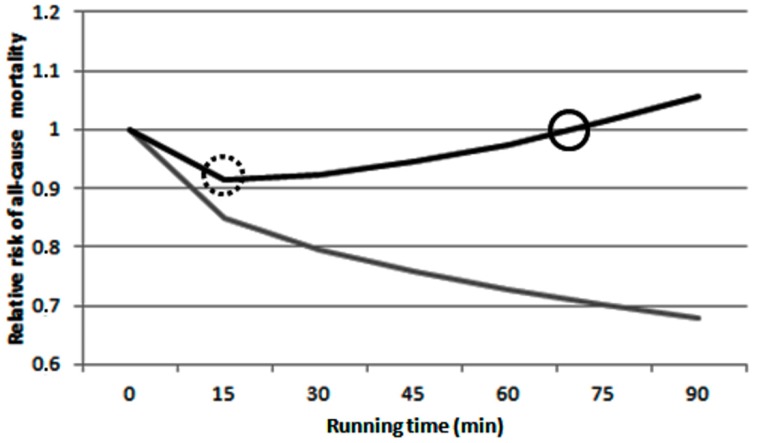
Relative risk of all-cause mortality according to exercise duration in the dirtiest (**black line**) and cleanest (**gray line**) cities. Dotted circle: tipping point; solid circle: break-even point.

The results of relative risk of all-cause mortality analysis are presented in Figure 4. It was observed a tipping point at 15 min of running, and a break-even point at 75 min of exercise only in the dirtiest cities. For cleanest cities, no relative risk transition points were observed.

## 4. Discussion

The present study compared the PM_10_ and PM_2.5_ inhaled dose during a traditional recommended 30 min daily exercise routine [3] at cities with distinct air pollution levels. Our main results suggest that exercise performed in the dirtiest cities might lead to about 37–66 times higher inhaled pollutants than in the cleanest cities, which could suppress the health benefits provided by aerobic exercise. 

It is interesting to highlight the significant differences in air pollution levels between the cleanest and dirtiest cities reported in the WHO database, being about 47 and 34 times higher in the dirtiest cities for PM_10_ and PM_2.5_, respectively. While cleanest cities are all presenting air pollution levels below the WHO guidelines, being considered ideal for health, the dirtiest cities present air pollution levels above the interim target 1. It has been reported in the literature that interim target 1 marks the air pollution level responsible for about 5% increase in short-term mortality over the air quality guidelines [19]. While cleanest cities are located in developed countries and are of relatively small size, the dirtiest cities are all located at underdeveloped countries, except the Chinese cities, and are of relatively larger size, similar to previously reported [20]. These observations, in accordance with others [21], suggest that geopolitical aspects, as economical status, high industrialization of peripheral countries, harmful energetic matrices, and cities planning are associated to populations’ air pollution exposure levels. This is in agreement with recent discussions raised in [22]. This is particularly important because several efforts have been made in order to improve public health, such as the stimulus for active transportation [23] and outdoor exercise practice [24,25]. Therefore, these findings suggest that different air pollution levels occasioned by socioeconomic status might influence the benefits from exercise practice, a recognized low-cost way to improve human health. 

Besides the intrinsic air PM concentrations, some exercise mechanisms might also enhance the inhaled PM. The influence of the increased VE during exercise on the inhaled PM has been demonstrated by Nyhan et al. [16] These authors demonstrated that the inhaled PM_2.5_ was higher in pedestrians and cyclists when compared to train and bus passengers, due to the higher metabolic demand of active transportation, which increases VE. Additionally, cyclists also presented a higher inhaled PM_2.5_ compared to pedestrians due the higher intensity of their transportation mode. Besides, Laeremans et al. [26] observed negative interaction effects of black carbon exposure during physical activity in lung function parameters obtained with spirometry, as forced expiratory volume in the first second and the Tiffeneau index., suggesting that inhalation level is an important marker of possible health deleterious effects from air pollution. Thus, our results suggest that the air pollution levels should be taken into account for physical activity recommendations. 

The results of the present study also revealed that only at the dirtiest cities, the air pollution would able to surpass exercise benefits over time, enhancing the relative risk of all-cause mortality. According with this analysis [18], the exercise benefits would continue increasing even after 90 min of exercise in the cleanest cities. On the other hand, the health benefits provided by aerobic exercised could be mitigate after only 15 min of exercise in the dirtiest cities. Furthermore, after 75 min, the additional exercise might cause adverse health effects due air pollution exposure. Tainio et al. [18] showed that to attain the tipping point in 30 min of exercise, the PM_2.5_ concentration needs to be of 95 µg·m^−3^, while for the break-even point attainment a PM_2.5_ concentration of 200 µg·m^−3^ would be necessary. Despite less than 1% of the cities in the WHO database presenting such air pollution levels, it is necessary to take into account that 30 min of physical activity is an extremely low duration when considering athletes and individuals working in active travel for long periods, such as postmen. Therefore, the establishment of exercise recommendations and prescriptions should be altered according to air pollution exposure.

It is important to consider some limitations of the present study. First, only the ten cleanest and dirtiest cities of the WHO database were analyzed. Despite these data do not represent all cities around the world, they provide an alert to the importance in controlling air pollution emissions. Further, as showed by Tainio et al. [18], the relative risk of all-cause mortality analysis takes into account only long-term health consequences of regular physical activity and chronic exposure to PM. Finally, using the annual average concentration provided in the WHO database, the large daily variation and the difference between places at the same city were not considered, what might be important for active transportation and physical activity practitioners. Thus, this analysis not considers the acute impacts of air pollution on health, which are important points to be considered in future publications, once acute effects are largely related to air pollution, mainly in susceptible groups. Secondly, only risks of mortality were taken into account, but not morbidity impact. Thus, this analysis might show even more alarming results if considering acute air pollution effects, such as cardiac events [13], and factors related to morbidity, such as asthma aggravation [27]. However, our data obtained with young healthy men should be cautiously analyzed and not be extrapoled to susceptible groups. Lastly, our incremental tests with 1-min stages might not be sufficient to VE to reach steady-state representing exactly 65% of the VO_2_max. Thus, real VE values might be even larger, which would lead to even more worrying air pollutants inhalation. 

## 5. Conclusions

In conclusion, our data suggest that a traditional aerobic training session of 30 min of moderate exercise could produce high levels of pollutant inhalation when performed in the dirtiest cities. Considering that pollutants inhalation is responsible for several adverse health effects from air pollution exposure, this augmentation in inhaled dose during exercise might trigger a rise in air pollution’s deleterious effects when exercise is performed in high polluted environments. Thereby, it might be important that future exercise guidelines consider environmental characteristics, emphasizing air pollution, to optimize benefits and to avoid health damages.

## Figures and Tables

**Table 1:** Ventilation at rest and exercise (*n* = 116).

	Rest	Exercise (65% VO_2_max)
VE (L·min^−1^)	8.1 ± 1.2	62.8 ± 10.1
VE_TOTAL_ (L)	243.0 ± 30.3	1883.1 ± 301.7

Values are means ± standard deviations. VE: ventilation rate in liters per minute; VE_TOTAL_: total ventilation for 30 min of each situation in liters.
